# Computational Analysis of HLA-presentation of Non-synonymous Recipient Mismatches Indicates Effect on the Risk of Chronic Graft-vs.-Host Disease After Allogeneic HSCT

**DOI:** 10.3389/fimmu.2019.01625

**Published:** 2019-07-16

**Authors:** Jarmo Ritari, Kati Hyvärinen, Satu Koskela, Riitta Niittyvuopio, Anne Nihtinen, Urpu Salmenniemi, Mervi Putkonen, Liisa Volin, Tony Kwan, Tomi Pastinen, Maija Itälä-Remes, Jukka Partanen

**Affiliations:** ^1^Finnish Red Cross Blood Service, Helsinki, Finland; ^2^Stem Cell Transplantation Unit, Department of Hematology, Comprehensive Cancer Center, Helsinki University Hospital, Helsinki, Finland; ^3^Stem Cell Transplantation Unit, Division of Medicine, Department of Hematology, Turku University Hospital, Turku, Finland; ^4^Department of Human Genetics, McGill University and Genome Quebec Innovation Centre, McGill University, Montreal, QC, Canada; ^5^Center for Pediatric Genomic Medicine, Children's Mercy, Kansas City, MO, United States

**Keywords:** whole-exome sequencing, HSCT, HLA, minor histocompatibility antigen, genomics, graft-vs.-host

## Abstract

Genetic mismatches in protein coding genes between allogeneic hematopoietic stem cell transplantation (allo-HSCT) recipient and donor can elicit an alloimmunity response via peptides presented by the recipient HLA receptors as minor histocompatibility antigens (mHAs). While the impact of individual mHAs on allo-HSCT outcome such as graft-vs.-host and graft-vs.-leukemia effects has been demonstrated, it is likely that established mHAs constitute only a small fraction of all immunogenic non-synonymous variants. In the present study, we have analyzed the genetic mismatching in 157 exome-sequenced sibling allo-HSCT pairs to evaluate the significance of polymorphic HLA class I associated peptides on clinical outcome. We applied computational mismatch estimation approaches based on experimentally verified HLA ligands available in public repositories, published mHAs, and predicted HLA-peptide affinites, and analyzed their associations with chronic graft-vs.-host disease (cGvHD) grades. We found that higher estimated recipient mismatching consistently increased the risk of severe cGvHD, suggesting that HLA-presented mismatching influences the likelihood of long-term complications in the patient. Furthermore, computational approaches focusing on estimation of HLA-presentation instead of all non-synonymous mismatches indiscriminately may be beneficial for analysis sensitivity and could help identify novel mHAs.

## Introduction

Allogeneic hematopoietic stem cell transplantation (allo-HSCT) presents a potentially curative treatment for a variety of malignant diseases and other serious disorders of the blood and hematopoietic system. The human leukocyte antigen (HLA) allele matching across several loci between the donor and recipient is a prerequisite for allo-HSCT to avoid lethal alloimmunity complications where the grafted T-cells mount an immune response against healthy recipient tissues (1). However, despite comprehensive HLA identity and advances in immunosuppressive medication, the graft-vs.-host disease (GvHD) remains a major cause of morbidity and mortality (2). On the other hand, alloreactivity directed against leukemic cells via the graft-vs.-leukemia (GvL) effect (3) is required for elimination of residual malignancy and curing the primary disease.

Following a HLA-matched allo-HSCT, the T-cell mediated alloimmunity is initiated mainly by non-HLA genetic differences encoding protein-level polymorphisms known as minor histocompatibility antigens (mHAs) (4). Common coding region genetic variability such as splicing variants (5), gene deletions (6), and non-synonymous single nucleotide variants (7) as well as *de novo* somatic mutations (8) can produce peptides that are presented by the HLA receptors on the cell surface and recognized by specific donor-derived T-cells as “non-self” epitopes. mHAs limited to the hematopoietic tissue are able elicit a highly specific GvL effect in allo-HSCT setting (9), while mHAs with broader and more varying expression profiles typically contribute to both GvL and GvHD (10). Since GvL can occur independent of GvHD, mHAs hold significant therapeutic potential for manipulating the alloimmunity (11). However, even though *in vitro* experimental analyses have identified several ligands for various alleles of different types of HLA receptors (12), only about 50 actual mHAs relevant for GvHD or GvL are currently known (13). Thus, extensive characterization of the mHA repertoire arising from germline or somatic genetic variability is expected to bring mHA targeted cell therapies closer to clinical application.

Due to the limited number of known mHAs, the extent of overall genome-wide non-synonymous mismatching has been proposed as a measure of alloreactivity potential in allo-HSCT recipients (14–16). In principle, separating the protein-coding mismatches by expression patterns could help assess the magnitude in and balance between GvHD and GvL. However, analysis of protein coding differences in related and unrelated allo-HSCTs has shown only weak association of estimated mismatching to the risk of severe acute GvHD (14). Further, in an alternative approach, computational prediction of HLA binding affinities of amino-acid altering genetic differences has shown a difference between related and unrelated allo-HSCTs (16), and has been reported to associate with GvHD risk (17). Studies analyzing the presence of known mismatched mHAs in allo-HSCT recipients have likewise identified small effects on GvHD risk and relapse-free survival (18–22). Given the various methodological approaches and that few of these associations remain statistically significant after multiple testing adjustment, further studies are warranted to investigate the graft alloimmunity capacity.

Here, we have analyzed genomic mismatching in 157 sibling allo-HSCT pairs to study the effects of HLA-presentation of non-synonymous variants on chronic GvHD. Based on previous reports, our hypothesis was that patients with severe cGvHD are more likely to harbor a higher number of mismatched HLA class I ligands than patients without cGvHD. Owing to the relatively small set of donor-recipient pairs in our study cohort, we have not considered acute GvHD since the number patients with severe form of this condition may not be adequate. Similarly, as the number of bone marrow expressed peptides is significantly smaller than peptides expressed in epithelial tissues, we have chosen not to focus on relapse or GvL. While the impact of predicted and experimental mHAs on GvHD have been studied previously by computational techniques, to our knowledge, existing databases of *in vitro* verified HLA ligands have not been employed in genomic studies of GvHD before. In contrast to mHAs, the relatively large amount of available experimental HLA ligands allows both statistical estimates with higher confidence and analyses in conjunction with other external data sets. Thus, comparison of this approach with established methods is needed to better understand alloreactivity and its computational modeling. To this end, we have carried out a computational analysis of class I HLA peptide binding affinity and immunogenicity potential, included epithelial protein expression data and enumerated the presence of experimentally verified HLA ligands and mHAs available in the public domain to evaluate the capability of these different approaches to estimating long-term alloreactivity capacity.

## Methods

### HSCT Pairs

The study cohort consisted of 157 Finnish HLA-matched sibling HSCT donors and recipients undergoing HSCT during the years 2001–2015 in two transplantation centers in Finland. The cohort and definitions of clinical outcomes have been described previously in detail (23). General characteristics of the cohort are given in Table 1. The study was approved by the Ethics Committees of Helsinki University Central Hospital and Turku University Central Hospital and the Finnish National Supervisory Authority for Welfare and Health (Valvira; https://www.valvira.fi/en/web/en). Informed and written consent was taken when possible, and in cases when it could not be asked (e.g., if the patient had deceased), a retrospective consent was given by the supervisory authority.

**Table 1:** General characteristics of the study cohort.

**Characteristic**	**Category**	**Value**
Graft type; number (percentage)	PB	118 (76.1)
	BM	37 (23.9)
Acute GvHD grade; number (percentage)	0	94 (59.9)
	1	28 (17.8)
	2	19 (12.1)
	3 & 4	16 (10.2)
Chronic GvHD grade; number (percentage)	No	77 (51.0)
	Limited	23 (15.2)
	Extensive	51 (33.8)
Relapse occurrence; number (percentage)	Yes	47 (30.1)
	No	109 (69.9)
Recipient-mismatched peptides; mean (95% CI)		872,895 (627,999–1,375,167)
Filtered recipient-mismatched peptides; mean (95% CI)		28,146 (17,925–44,979)
M1; mean (95% CI)		393 (88–889)
M2; mean (95% CI)		21.6 (3.0–47.1)
M3; mean (95% CI)		2.6 (0.0–10.1)
M4; mean (95% CI)		39.5 (2.0–172.6)

*For explanations of analysis approaches M1–M4 (see Table 2)*.

### HLA-Typing and Matching

The extent of HLA matching between the sibling donor-recipient pairs has varied over time during the 1990s and the 2000s. Depending on a given time period, HLA-C, and/or HLA-DQB1 genes were genotyped only in the pairs with an HLA mismatch in any of the HLA-A, -B, -DRB1 genes, conforming with the European Federation of Immunogenetics (EBI) guidelines at the time. Thus, not all pairs were initially guaranteed to match over the six HLA genes (HLA-A, -B, -C, -DRB1, -DQB1, DPB1). Hence, all pairs were re-typed with NGS technology in this study.

HLA typing was performed as described previously (24). Briefly, the alleles of HLA-A, -B, -C, -DQB1, and -DRB1 genes were identified at the resolution level of the first field (i.e., allele group) in the HLA Laboratory of the Finnish Red Cross Blood Service using rSSO-Luminex technology (Labtype, One Lambda, Inc., CA, USA) and PCR-SSP (Micro SSP™ Generic HLA Class I/II DNA Typing Trays, One Lambda, Inc.; Olerup SSP® genotyping, Olerup SSP AB, Stockholm, Sweden). Two pairs with 5/6 HLA-match were confirmed by sequencing at the second field resolution level (i.e., amino acid level) (AlleleSEQR PCR/Sequencing kits, Atria Genetics, Hayward, CA, USA) using ABI 3130xl genetic analyzer (Applied Biosystems, Thermo Fisher Scientific, MA, USA). The results were analyzed with Assign 3.5+ software (Conexio Genomics Pty Ltd, Fremantle, Australia). The FASTQ read data of all pairs were analyzed using the Omixon Explore program version 1.2.0 (Omixon, Budapest, Hungary) to assign alleles at third field resolution level (i.e., allelic level) for HLA-A, -B, -C, -DRB1, -DQB1, -DPB1.

### Exome Sequencing

Exome sequencing and quality control was performed as described previously (23). Briefly, a custom sequencing panel targeting the whole exome, full MHC region and active immunoregulatory regions was applied (25). The sequencing was performed with Illumina HiSeq 2000 instrument at the McGill Genome Center (McGill University, Montreal, Canada). The resulting reads were aligned against the human reference genome GRCh37/hg19 (25). Variant calling and quality filtering steps were carried out using the Genotype Analysis Tool Kit (GATK) v3.2–2 (26, 27), and further quality filtering was implemented by applying a hard cutoff on approximate read depth (DP) and genotype quality (GQ) parameter values obtained through comparing genotype similarities between technical replicates.

### Experimental HLA Ligand Data

HLA ligand assay data was downloaded from the Immune Epitope Database (IEDB) website (www.iedb.org) (12) in October 2016. Assay results were filtered to include only ligands that originated from *H. sapiens*, had qualitative measure of “positive” or “positive-high” and had HLA allele information available. Duplicated peptides were removed. Minor histocompatibility antigen data set was obtained from published literature (13). Peptides longer than 9 amino acids were transformed into 9-mer format by extracting all 9-mer frames. Duplicated entries were removed.

### Peptide Data Analyses

**Figure 1:**
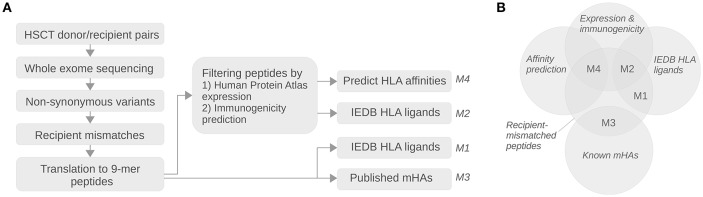
Schematic diagram of the analysis pipeline. **(A)** The vertical arrows in top-down direction in the diagram show the processing steps for translating whole exome sequencing data into recipient-mismatched peptide sets. The horizontal arrows in left-to-right direction show the analysis steps involved in sub-setting the mismatched peptides into sets relevant for alloreactivity; in the first step the peptides are filtered for epithelial expression and immunogenicity, and in the second step they are intersected with experimental HLA ligand databases or HLA affinity predictions according to each pair's HLA class I type. Finally, the obtained HLA-presented peptide count estimates from the four analysis approaches (labeled as M1–M4; Table 2) are examined for possible association with chronic GvHD. **(B)** Euler diagram showing the analysis methods M1–M4 as intersections between peptide sets.

The analysis pipeline is summarized in Figure 1. Variant positions annotated as locating in a protein coding region and having a missense, insertion, deletion, frameshift, or stop relative to the GRCh37 reference genome were extracted from the sample VCF files. The GRCh37.75 fasta-formatted coding sequences were downloaded from the Ensembl FTP server (ftp://ftp.ensembl.org/pub/release-75/fasta/homo_sapiens/cds/Homo_sapiens.GRCh37.cds.all.fa.gz) (28, 29) and filtered by including only entries starting with the canonical ATG codon and having a length of at least 50 nucleotides. The non-synonymous variants in each sample were mapped to the filtered GRCh37 reference coding sequences followed by translation of the generated individual transcriptome of each subject into protein sequences. The *in silico* proteomes between each HSCT donor-recipient pair were compared to exclude identical protein sequences. Proteins with at least one amino acid difference were split into 9-mer peptides from all frames, and peptides unique to the recipient were extracted and included in further analyses.

HLA class I immunogenicity prediction on the peptides was performed with the IEDB (12) Immunogenicity-1.1 tool (30) without HLA type specification. Peptides with immunogenicity score >0.2 were selected for further analysis.

**Table 2:** Computational approaches for estimating HSCT alloreactivity.

**Method label**	**Description**
M1	The number of recipient-mismatched peptides shared with experimental 9-mer HLA class I ligands from IEDB
M2	The number of recipient-mismatched peptides filtered by immunogenicity prediction and HPA data and shared with experimental 9-mer HLA class I ligands from IEDB
M3	The number of recipient-mismatched peptides shared with known 9-mer HLA class I mHAs
M4	The number of the recipient-mismatched peptides filtered by immunogenicity prediction and HPA data having HLA class I affinity prediction consensus rank 4 or less

Expression levels for the filtered peptides were acquired from the Human Protein Atlas (HPA) database (31) v18/Ensembl v88.38. The data were filtered to include only proteins for which reliability was classified as supportive in skin, intestine, lung, liver or bone marrow tissue. The HPA data were mapped to the peptide sequence data based on Ensembl transcript and gene IDs.

The HLA-A and -B receptor binding affinity predictions to the unique peptides of the HSCT recipients were performed with the IEDB tool *predict_binding.py* v2.17 using the *IEDB_recommended* option which combines the results of multiple prediction algorithms (32–37). The consensus percentile rank with cutoff values of ≤ 4 or ≤ 6 were used in further analyses as a measure of number of high-affinity peptides in each recipient.

The sets of recipient-specific peptides obtained as described above were intersected with the filtered IEDB HLA ligand peptide sequences and published mHA peptide sequences to calculate the numbers of these HLA ligands in each recipient. Table 2 summarizes the four methods for estimating alloreactivity based on the above analyses of the peptide sets.

### Statistical Methods

Statistical tests were carried out in R v3.4.4 (38) with custom scripts. Association between estimated ligand count measures and the chronic GvHD grades “none” vs. “extensive” was tested with logistic regression using the R function glm. *P*-values were calculated using the default Wald test. Three variables that could confound the estimates of the number of HLA-presented peptides were included as covariates in the regression analyses. The rationale was that rare HLA types may not be as well-covered as more common ones in the HLA ligand databases or HLA binding affinity training data, and can thus lead to under-representation of ligands in these types. Similarly, the total number of mismatches could mask the possible effect of HLA-presented peptides. Thus, the used covariates were: the sum of cohort frequencies of unique HLA types present in each recipient, the number of unique HLA types in each recipient, and the total number of mismatched peptides in each recipient. Furthermore, transplantation year, donor age and transplant direction (female-to-male vs. others) and the extent of matching over six HLA genes (HLA-A, -B, -C, -DRB1, -DQB1, -DPB1) for each HSCT pair were also included as covariates as these can be relevant for allo-HSCT outcome (22, 39). Each numerical predictor variable was centered and scaled. Benjamini–Hochberg adjusted *p*-value of <0.05 was considered significant. Data management and plotting were carried out using the R libraries *tidyverse* v1.2.1 (40), *ggpubr* v0.1.6 (41), *seqminer* v6.0 (42), *data.table* (43), and *Biostrings* v2.42.1 (44).

### Code Availability

The analysis code is available in GitHub (https://github.com/FRCBS/HSCT-peptide).

## Results

The results of the exome sequencing genotype calling and quality filtering steps have been described previously (23). To summarize, the obtained mean depth of read mapping coverage was 32.8 with standard deviation of 7.0 over all the samples. The donors had in total 468,426 quality filtered variants and the recipients had 470,135. The numbers of all 9-mer peptides generated from the recipient-mismatched non-synonymous variants had a mean of 872,895, standard deviation of 179,627, minimum of 281,009, and maximum of 1,583,366. Numbers of 9-mer peptides filtered for immunogenicity and HPA data had a mean of 28,146, standard deviation of 6,205, minimum of 8,523, and maximum of 51,425. On average, the number of unique peptides per sample with predicted high binding affinity was 39.5, ranging between 0 and 302 with a standard deviation of 46.9. Further details are given in Table 1.

HLA matching measured as the number of identical alleles between the donor-recipient pairs over the six genes (HLA-A, -B, -C, -DRB1, -DQB1, -DPB1) had a mean of 11.73 and a standard deviation of 1.31. 147, 3 2, and 5 patients had 12, 11, 10, and 9 or less matched alleles, respectively. To evaluate the HLA allele overlap between our data and the utilized external data sources, the HLA class I allele representation in the IEDB and mHA data sets was explored. For HLA-A, 96.8% of alleles in our cohort were found in the IEDB data and 78.7% in the mHA data. For HLA-B, the figures were 99.8 and 31.8%, respectively. 89.5% of HLA-C alleles were found in the IEDB data, and HLA-C was not represented in the mHA data.

**Figure 2:**
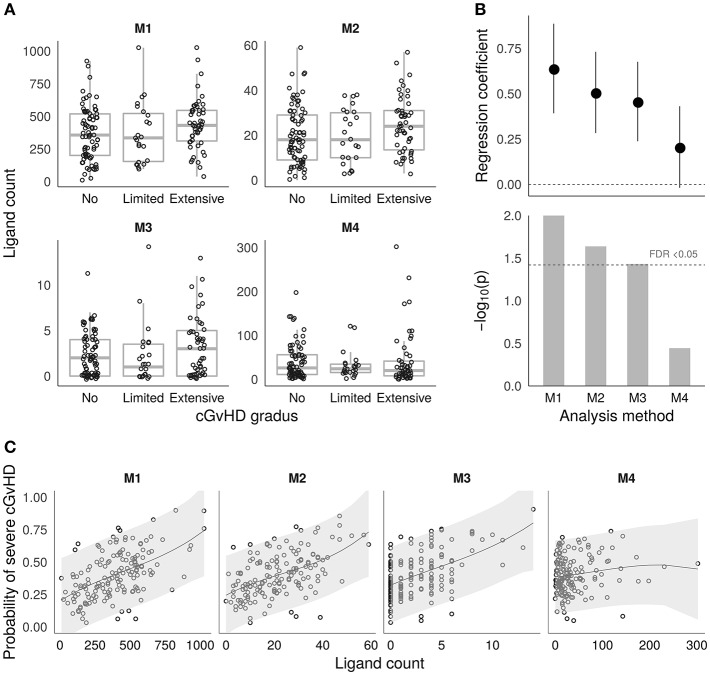
Alloimmunity estimates and associations with chronic GvHD. **(A)** Boxplots showing the distributions of the four different HLA ligand estimates (M1–M4) based on recipient-mismatched peptides (see the Methods section and Table 2 for a detailed description). The y-axis shows the estimated ligand count, and the x-axis shows the cGvHD grades. **(B)** Logistic regression coefficient estimates with ± 1 S.E. (top) and *p*-values (bottom) from the models testing the associations for cGvHD grades “no” (*n* = 77) vs. “extensive” (*n* = 51) in the four methods M1–M4. The coefficients are calculated based on scaled and centered ligand count values to make them comparable between M1–M4. The FDR < 0.05 threshold is shown by the dashed horizontal line in the lower panel. **(C)** Estimated probabilities for severe chronic GvHD vs. the number of mismatched ligands as given by the fitted logistic regression models for M1–M4. The shaded areas visualize the 95% confidence intervals for prediction.

Based on the sets of recipient-mismatched peptides, four approaches to estimating cGvHD related alloreactivity in sibling HSCT were evaluated as given in Table 2. The estimated ligand counts as given by the four approaches (termed M1–M4) are shown by the boxplots in Figure 2A. The results of logistic regression analysis between the estimates and cGvHD grades “no” vs. “extensive” (*n* = 77 and *n* = 51, respectively) are summarized by Figure 2B. The logistic regression model probabilities for severe cGvHD vs. recipient-mismatched ligand counts are shown by Figure 2C. Full logistic regression results are given in Table 3. The obtained nominal *p*-values for methods M1–M4 were 0.00976, 0.0234, 0.0366, and 0.36, respectively. After Benjamini–Hochberg FDR adjustment the values were 0.04, 0.046, 0.0493, and 0.36, respectively. Thus, M1–M3 remained significant (FDR < 0.05). The obtained regression coefficients (log odds) and their 95% confidence intervals, respectively, were 0.639 (0.392–0.886), 0.507 (0.283–0.731), 0.457 (0.238–0.676), and 0.206 (−0.019–0.431), Thus, the methods M1–M4 were all consistent in their positive effect direction (Figure 2B). To evaluate the robustness of the M4 approach to parameter value selection, a less stringent cutoff value of ≤ 6 was also used. This did not significantly change the result as the obtained regression coefficient was 0.196.

**Table 3:** Logistic regression results for chronic GvHD by analysis methods M1–M4.

**Method**	**Variable**	**Estimate**	**Std. deviation**	***Z*-value**	***P*-value**
M1	(Intercept)	−2.97	3.54	−0.84	0.4
	Ligand count	0.64	0.25	2.58	0.01
	Number of mismatched peptides^a^	−0.06	0.22	−0.28	0.78
	Sum of HLA frequencies	−0.44	0.25	−1.78	0.07
	Tr direction	−0.89	0.48	−1.87	0.06
	Donor age	0.33	0.21	1.54	0.12
	Number of unique HLAs	0.13	0.21	0.62	0.53
	Tr year	0.13	0.23	0.59	0.56
	HLA matching	0.27	0.29	0.93	0.35
M2	(Intercept)	−3.19	3.43	−0.93	0.35
	Ligand count	0.51	0.22	2.27	0.02
	Number of mismatched peptides^b^	0.02	0.21	0.1	0.92
	Sum of HLA frequencies	−0.38	0.24	−1.6	0.11
	Tr direction	−0.87	0.47	−1.85	0.06
	Donor age	0.27	0.21	1.27	0.2
	Number of unique HLAs	0.14	0.21	0.65	0.52
	Tr year	0.11	0.23	0.5	0.62
	HLA matching	0.29	0.29	1.02	0.31
M3	(Intercept)	−3.11	3.62	−0.86	0.39
	Ligand count	0.46	0.22	2.09	0.04
	Number of mismatched peptides^a^	0.04	0.2	0.2	0.85
	Sum of HLA frequencies	−0.32	0.23	−1.4	0.16
	Tr direction	−0.8	0.46	−1.73	0.08
	Donor age	0.34	0.21	1.6	0.11
	Number of unique HLAs	0.18	0.21	0.84	0.4
	Tr year	0.13	0.22	0.6	0.55
	HLA matching	0.28	0.3	0.93	0.35
M4	(Intercept)	−2.8	3.58	−0.78	0.43
	Ligand count	0.21	0.23	0.92	0.36
	Number of mismatched peptides^b^	0.11	0.2	0.55	0.59
	Sum of HLA frequencies	−0.21	0.21	−1	0.32
	Tr direction	−0.76	0.46	−1.65	0.1
	Donor age	0.32	0.21	1.56	0.12
	Number of unique HLAs	0.26	0.26	1	0.32
	Tr year	0.02	0.22	0.11	0.92
	HLA matching	0.25	0.3	0.84	0.4

a The numbers of all recipient-mismatched peptides.

b The numbers of recipient-mismatched peptides filtered by immunogenicity and expression.

## Discussion

Collectively, the results of the present study assessing the alloimmunity capacity in a sibling allo-HSCT setting suggest that the risk of extensive chronic GvHD is increased by a higher degree of recipient mismatching. Even though the observed effect was relatively weak, all four of the approaches applied to estimating the alloimmunity capacity were in agreement with regard to their effect direction, supporting the authenticity of the association with chronic GvHD and suggesting that these methods are able to measure relevant properties of recipient-mismatching peptides. This result is also consistent with studies analyzing the presence of sets of known recipient-mismatched mHAs in chronic GvHD (18, 21). However, a study by Martin et al. using the total sum of mismatches in coding variants as a measure of alloimmunity potential (14) reported an association for acute GvHD in sibling allo-HSCT, but found no association for chronic GvHD. This discrepancy could be due to differences in methodology, as in our study we focused solely on HLA-presentation of mismatching peptides rather than all non-synonymous mismatches. Synonymous variants in general have been recognized to affect gene expression through codon usage bias (45) and in this way may contribute to mismatching in allo-HSCT as well. In terms of disease mechanisms, it is established that the acute form of GvHD is triggered by strong cytokine storm as a result of leakage of lipopolysaccharides of commensal microbes from the intestinal lumen (46). Moreover, autosomal mHAs were not found to associate with acute GvHD, except for Y-chromosomal mHAs in female-to-male HSCT (22). Hence, the role of alloimmune differences between the donor and recipient may be secondary for acute GvHD, but could influence the risk for autoimmune-like chronic GvHD.

Out of the four methods applied for assessing the alloimmunity capacity, the *in silico* prediction of HLA peptide binding affinity and immunogenicity showed the weakest association. Although prediction of HLA binding for peptides originating from within known mHA-producing genes has been shown to allow discovering more potential mHAs in the same genes and correlate with GvHD risk (20), applied genome-wide, the computational prediction method did not significantly associate with clinical outcome. This could be due to the fact that the process of peptide cleavage and the formation of HLA-peptide complex are still incompletely understood and cannot be reliably modeled from sequence alone (47, 48). However, since in our analysis we did not model proteasomal cleavage or TAP transport independently of HLA affinity, their contribution to the overall prediction capacity of HLA presentation of peptides could not be assessed. In a genome-wide application, this approach could lead to a relatively high number of false positives or low sensitivity, and thus may not be ideal for assessing total alloimmunity capacity. Also, the choice of an inclusion threshold value for predicted high-affinity peptides could affect the analysis result, albeit our analysis with lower stringency threshold suggests that the approach is relatively robust for the choice of threshold.

Moreover, we were not able to find advantage in limiting the pool of analyzed recipient-mismatched and IEDB matched peptides to computationally predicted immunogenic and hematopoietic and epithelial tissue expressed molecules. Similarly to current methods of HLA affinity prediction, the immunogenicity algorithms may not be accurate enough for genome-wide application despite that these computational tools have proven to be useful when combined with an experimental proteogenomics approach that specifically targets HLA-associated peptides (47). On the other hand, the accuracy of immunogenicity estimate may benefit from masking of certain amino acid positions in particular alleles which was not implemented in our filtering step. Furthermore, the observation that utilizing all available IEDB ligands together with total recipient-mismatched peptides provided the best estimate suggests that there may be numerous, presently unknown mHAs relevant for alloimmunity (49).

While the method employing known mHAs showed an effect to the same direction as the full IEDB HLA ligand based approach, the effect was clearly smaller, which could be due to low numbers of mHA peptides in the set and limited to a few HLA alleles, reducing the signal-to-noise ratio and leading to lower statistical power. Our study is also limited by restricting the analysis exclusively to 9-mer peptides whereby it cannot address the effect of ligands presented by HLA class II receptors such as HLA-DR, -DB, and -DP (13). Thus, expanding the peptide length repertoire could improve particularly the results of the mHA based analysis, provided that sufficient HLA class II mHA data are available. Furthermore, the results we obtained for chronic GvHD may not be directly comparable with non-European populations owing both to our study cohort and to the IEDB and mHA data collections' focus on ligands from European subjects. Allele frequencies differing between populations (50) may impact the likelihood of finding matches in HLA ligand data sets.

In summary, the effects observed by us and others in similar studies emphasize the need for a comprehensive meta-analysis and large cohorts to gain further insight into the impact of non-HLA mismatches in allo-HSCT. In particular, it is essential to expand the knowledge of the mHA repertoire for the design of novel treatments and diagnostic tools for allo-HSCT complications. In this respect, it may be beneficial to employ computational approaches making use of available experimental HLA ligand data.

## Data Availability

The datasets for this manuscript are not publicly available because: they contain unique identifying information of the patients. Requests to access the datasets should be directed to JP, jukka.partanen@bloodservice.fi.

## Ethics Statement

The study was approved by the Ethics Committees of Helsinki University Central Hospital and Turku University Central Hospital and the Finnish National Supervisory Authority for Welfare and Health (https://www.valvira.fi/en/web/en).

## Author Contributions

JP and JR designed the study. SK managed the patient DNA samples and provided expertise on HLA typing. MP, AN, RN, MI-R, US, and LV managed and collected the clinical data on patients and provided clinical expertise. TK and TP performed the sequencing. KH, JR, and TK preprocessed the data. JR analyzed the data and wrote the manuscript.

### Conflict of Interest Statement

The authors declare that the research was conducted in the absence of any commercial or financial relationships that could be construed as a potential conflict of interest.
